# EphA2 Expression in Bone Sarcomas: Bioinformatic Analyses and Preclinical Characterization in Patient-Derived Models of Osteosarcoma, Ewing’s Sarcoma and Chondrosarcoma

**DOI:** 10.3390/cells10112893

**Published:** 2021-10-26

**Authors:** Giorgia Giordano, Alessandra Merlini, Giulio Ferrero, Giulia Mesiano, Erika Fiorino, Silvia Brusco, Maria Laura Centomo, Valeria Leuci, Lorenzo D’Ambrosio, Massimo Aglietta, Dario Sangiolo, Giovanni Grignani, Ymera Pignochino

**Affiliations:** 1 Candiolo Cancer Institute, FPO–IRCCS Str. Prov.le 142, Km 3.95, 10060 Candiolo, Italy; giorgia.giordano@ircc.it (G.G.); giulia.mesiano@ircc.it (G.M.); erika.fiorino@ircc.it (E.F.); silvia.brusco@ircc.it (S.B.); marialaura.centomo@ircc.it (M.L.C.); valeria.leuci@ircc.it (V.L.); LDambrosio@asl.at.it (L.D.); massimo.aglietta@ircc.it (M.A.); dario.sangiolo@ircc.it (D.S.); giovanni.grignani@ircc.it (G.G.); ymera.pignochino@ircc.it (Y.P.); 2 Department of Oncology, University of Torino, 10124 Torino, Italy; 3 Department of Clinical and Biological Sciences, University of Torino, 10124 Torino, Italy; giulio.ferrero@unito.it; 4 Department of Computer Science, University of Torino, 10124 Torino, Italy; 5 Cardinal Massaia Hospital, 14100 Asti, Italy

**Keywords:** target therapies, bioinformatics, EphA2, osteosarcoma, Ewing’s sarcoma, chondrosarcoma

## Abstract

Bone sarcomas are a group of heterogeneous malignant mesenchymal tumors. Complete surgical resection is still the cornerstone of treatment, but, in the advanced/unresectable setting, their management remains challenging and not significantly improved by target- and immuno-therapies. We focused on the tyrosine kinase Eph type-A receptor-2 (EphA2), a key oncoprotein implicated in self-renewal, angiogenesis, and metastasis, in several solid tumors and thus representing a novel potential therapeutic target. Aiming at better characterizing its expression throughout the main bone sarcoma histotypes, we investigated EPHA2 expression in the Cancer Cell Lines Encyclopedia and in public datasets with clinical annotations. looking for correlations with molecular, histopathological and patients’ features and clinical outcomes in a total of 232 osteosarcomas, 197 Ewing’s sarcomas, and 102 chondrosarcomas. We observed EPHA2 expression in bone sarcoma cell lines. We demonstrated higher EPHA2 expression in tumor tissues when compared to normal counterparts. A significant correlation was found between EPHA2 expression and Huvos grade (osteosarcoma) and with worse overall survival (dedifferentiated chondrosarcoma). Next, we characterized EPHA2 expression and activation in bone sarcoma primary tissues and in patient-derived xenografts generated in our laboratory to verify their reliability as in vivo models of osteosarcoma, Ewing’s sarcoma and chondrosarcoma. Furthermore, for the first time, we demonstrated EPHA2 expression in chondrosarcoma, suggesting its potential key role in this histotype. Indeed, we observed a significant dose-dependent antitumor effect of the EphA2-inhibitor ALW-II-41-27 in patient-derived in vitro models. In conclusion, EphA2 targeting represents a promising novel therapeutic strategy against bone sarcomas.

## 1. Introduction

Bone sarcomas are a rare and heterogeneous group of malignant mesenchymal primary tumors originating from the osseous tissue, representing less than 1% of all malignancies. In particular, osteosarcoma and Ewing’s sarcoma occur mainly in adolescents and young adults, while chondrosarcoma has a peak incidence in the seventh decade [1]. The addition of chemotherapy to surgical excision in osteosarcoma and Ewing’s sarcoma greatly improved the outcome of patients affected by localized disease. Despite optimal treatment, 30–40% of patients still die of the disease due to poor histological response to therapy, disease relapse and onset of multiple metastases [1]. Several target therapies, conventional chemotherapy and immunotherapeutic strategies have been investigated, but besides mifamurtide no other innovative therapy has been approved for the clinical treatment of osteosarcoma in the last decade [2]. The identification of novel molecular targets will be instrumental for overcoming the current impasse in the treatment of relapsing and non-resectable disease presentations. Indeed, multikinase inhibitors with a specific antiangiogenic activity in osteosarcoma [3,4,5], a novel inhibitor of transcriptional-promoting activity of ETS family transcription factors in sarcomas [6], and inhibitors of IDH1/2 activity in chondrosarcomas [7,8], have displayed some clinical benefits, but without exceeding a progression-free survival in the range of six months. These results underpin the need to continue exploring innovative avenues in the quest for key targets through which novel therapies could hijack tumor progression and metastasis. In this view, molecules involved in cell migration and tumor metastasis are of particular interest. 

Among these molecules, erythropoietin-producing hepatocellular (Eph) tyrosine kinase receptors are the largest known family of tyrosine kinase receptors. They mediate bi-directional signals between adjacent cells. Eph receptors modulate cytoskeleton dynamics, cell adhesion and cell motility. Indeed, they orchestrate cell movements during several processes (mainly during embryo development), such as gastrulation, segmentation, angiogenesis, axonal path finding, and neural crest cell migration [9,10,11]. In this protein family, Eph type-A receptor 2 (EphA2) has been identified as a driver oncoprotein in the pathogenesis of several tumors, including bone sarcomas, being implicated in the acquisition of self-renewal (stem cell-like) properties [12] and metastasis [13]. In these tumor-promoting contexts, EphA2 activation relies on the so-called non-canonical EphA2 pathway, which is characterized by phosphorylation at serine 897 by pro-tumorigenic kinases, such as PKA, RSK kinases and Akt [14,15,16]. Its “canonical” activation through ligand binding, tyrosine kinase activity and tyrosine autophosphorylation has been implicated in EphA2 activation in physiological conditions [11,17,18]. EphA2 is highly expressed in a variety of tissues and processes during embryo development, as with other Eph protein family members [9,10,11]. However, in adult tissues, its expression is low and mostly limited to epithelia, with a high proportion of dividing cells [17,18]. This could be an important prerequisite for selecting EphA2 as a potential therapeutic target. 

In our previous work, we demonstrated that combined pazopanib + trametinib treatment could significantly reduce EphA2 expression in osteosarcoma cell lines and that this was possibly a relevant mechanism of response to the combination treatment [19].

In this study, we aim to broaden our validation of EphA2 as a therapeutic target across the bone sarcoma family, particularly in osteoblastic osteosarcoma, Ewing’s sarcoma and conventional chondrosarcoma, and at establishing bone sarcoma models, tailored according to patients’ tumor characteristics. We propose an in silico investigation of EPHA2 expression in bone sarcoma cell lines and in publicly available datasets from bone sarcoma samples of case series, showing the involvement of EPHA2 in the three main aforementioned bone sarcoma histotypes. We also describe the correspondence among primary tumor tissues and patient-derived xenograft (PDXs) models, focusing on EphA2 expression. To our knowledge, EphA2 had not yet been investigated in chondrosarcoma. This brings chondrosarcoma to the fore alongside osteosarcoma and Ewing’s sarcoma as a potential preclinical and clinical field of study for anti-EphA2 targeting and immunotherapeutic strategies, which are already ongoing, or with preliminary data in several cancer types (NCT02252211, NCT04180371, NCT02575261) [20,21,22,23]. 

Indeed, we showed that EphA2 targeting with the small molecule inhibitor ALW-II-41-27 resulted in marked reduction in cell growth and cell viability in four in vitro models, which we derived from primary osteosarcoma, Ewing’s sarcoma and chondrosarcoma tumor tissues.

Finally, our established PDX models could serve as a functional validation of EphA2’s relevance in the progression of osteosarcoma, Ewing’s sarcoma and chondrosarcoma for further testing EphA2-targeting strategies in vivo, in different histotypes and at various disease stages (i.e., localized vs. metastatic).

## 2. Materials and Methods

### 2.1. Bioinformatics Analyses

The data on the EPHA2 expression levels in the cell lines were retrieved from the Cancer Cell Line Encyclopedia (CCLE) [24]. Specifically, the Z-score-normalized EPHA2 levels were retrieved from the CbioPortal webtool [25] using the track named “Cancer Cell Line Encyclopedia (Broad, 2019)”. R v4.0.3 and shinyGEO were used to analyze the EPHA2 expression levels in relation to patients’ clinical features in 88 osteosarcoma samples from the TARGET-OS project data retrieved from the NCI Genomic Data Commons and from three public gene expression experiments deposited in Gene Expression Omnibus (GEO): GSE39055 (37 osteosarcoma samples), GSE21257 (53 osteosarcoma samples). For the TARGET-OS data, the analysis of the relationship between EPHA2 levels and sex was computed using Wilcoxon-Rank sum Test (Wilcoxon.test R function) while the overall survival analysis was performed using the survival R package v3.12. For the analysis of the GEO datasets, shinyGEO was applied using the Differential Expression Analysis module for comparing the EPHA2 levels between patient groups based on specific covariates (sex, Huvos grade, and survival/recurrence status), while the Survival Analysis module was applied for the overall survival and relapse-free survival analyses. ShinyGEO was also used to analyze the relationship between the EPHA2 levels and clinical features of 246 Ewing’s sarcoma samples from GSE34620 (117 ES samples), GSE63155 (46 ES samples), GSE17674 (44 ES samples) and GSE63156 (39 ES samples). The data on the EPHA2 expression in 102 chondrosarcoma samples were retrieved from E-MTAB-7264. These microarray data were preprocessed and normalized using the affy v1.70 R package in default settings, while Wilcoxon-Rank sum was used for statistical testing.

### 2.2. Establishment of Patient-Derived Xenograft Models

Patient-derived xenografts (PDXs) were established according to protocols approved by both human and animal Institutional Review Boards committee and the Italian Ministry of Health (Prot. 21635.18, Aut.Min.834/2019-PR). The patients’ characteristics are listed in Table 1. The patients provided signed informed consent; fresh tumor specimens obtained from surgical samples were selected by our local pathologist, who prepared vital tumor mass and surrounding/flanking normal tissues (healthy control tissue). Tumors were fragmented with a scalpel into 0.4 cm × 0.4 cm pieces and then implanted subcutaneously into the right flank of 7-week old NSG (NOD-SCID IL2R gamma null) mice (Charles River, Calco, Italy). Tumor engraftment and growth was monitored biweekly and the xenografts were explanted when a maximum diameter of 1.5 cm was reached. The explanted xenograft was then re-implanted in two further generations of mice and finally explanted and processed for molecular and histological analyses. 

### 2.3. RNA Extraction and Quantitative Real-Time PCR

The total RNA, including small non-coding RNAs, was extracted from snap-frozen primary tumor tissue and normal counterparts upon homogenization by using Promega a Maxwell RSC miRNA tissue Kit and a Maxwell RSC Instrument (Promega Italia, MI, Italy). RNA quality and concentration were checked by DS 11+ Spectrophotometer (Denovix Inc, Wilmington, DE, USA). Starting from 1 µg of total RNA, Superscript VILO IV Master Mix (Life Technologies Italia, Monza, Italy) was used to obtain the cDNA. Quantitative real-time PCR (qRT-PCR) was performed with TaqMan Fast Advanced Mastermix (Life Technologies Italia, Monza, Italy) and Taqman probes (EphA2: Hs01072272_m1 and ACTB: Hs99999903_m1) with an ABI PRISM 7900HT System (Thermo Fisher Scientific, Whaltam, MA, USA). The expression data were normalized to the geometric mean of housekeeping genes and log2 fold change in tumor tissue with respect to normal tissue control was calculated with the formula: 2^−ddct^. The experiments were dperformed in triplicates; the mean and SEM were calculated by using GraphPad Prism 8 (GraphPad Software, San Diego, CA, USA).

### 2.4. Western Blot

The total protein extracts were obtained from six different PDX-derived fresh-frozen tumor samples. After mechanical disaggregation in the presence of Lysis Buffer 6 (R&D Systems, Biotechne, Minneapolis, MN USA), western blotting was performed as previously described [19]. Briefly, the protein concentrations were determined using a bicinchoninic acid assay (BCA Protein Assay, Thermo Fisher Scientific, Whaltam, MA, USA), and the absorbance levels were measured using a BioPhotometer (Eppendorf, Hamburg, Germany). Samples of 10 to 30 µg were resolved by electrophoresis on 4–15% mini-PROTEAN^®^TGX™ PreCast gels (Bio-Rad Laboratories, Hercules, CA, USA) and then transferred to a nitrocellulose membrane via the Trans-Blot^®^ Turbo™ Transfer System (Bio-Rad Laboratories). The blocking of non-specific sites was performed with BSA 10% for 1 h at room temperature. Next, the membranes were incubated overnight at 4 °C with primary antibodies: Phospho-EphA2 (Ser897) (D9A1) Rabbit mAb #6347, EphA2 (D4A2) XP^®^ Rabbit mAb#6997 and β-Actin (13E5) Rabbit mAb #4970 from Cell Signalling Technologies (Euroclone, Pecco, Italy). Specific signals were visualized with HRP–conjugated secondary antibodies (Jackson Immuno Research Laboratories, West Grove, PA, USA) and detected using the Bio-Rad Chemidoc™ Touch Imaging System (Bio-Rad Laboratories) following exposure to Clarity™ Western ECL substrate (Bio-Rad Laboratories).

### 2.5. Immunohistochemistry

The immunohistochemistry (IHC) for EphA2 was performed manually according to standard procedure, on paraffin-embedded PDX tumor tissues. The antigen retrieval was performed with 15-min sub-boiling citrate buffer (pH = 6). Next, a peroxidase blockade was carried out with serum-free Dako blocking reagent (Agilent Technologies, Inc., Santa Clara, CA, USA). The primary antibody for EphA2 staining was D4A2 XP^®^ Rabbit mAb #6997 (Cell Signaling Technologies, Danvers, MA, USA) diluted 1:200 and incubated overnight at 4 °C. Staining was obtained with Dako EnVision+ System- HRP Labelled Polymer Anti-Rabbit (Agilent Technologies, Inc., Santa Clara, CA, USA) followed by 3,3′-Diaminobenzidine as the standard chromogen and counter-staining with hematoxylin. Slide fixation was performed with mounting medium and observation under an optical microscope (Leica DM750, Leica Biosystems, Buccinasco, MI, Italy) equipped with a Leica ICC50W camera (Leica Biosystems).

### 2.6. Primary Cell Lines

The primary cell lines were obtained from the corresponding fresh or frozen tumor samples. Tumor samples (approximately 10 mm^3^ for each sample) were dissociated with a surgical scalpel and subsequently reduced to single-cell suspension using a Tumor Dissociation Kit, human (Miltenyi Biotech, Bologna, Italy) and GentleMACS™ Octo Dissociator (Miltenyi Biotech). Cells were cultured in monolayer condition in the presence of Gibco BRL KO DMEM F12 (Thermo Fisher Scientific), with 10% fetal bovine serum (Euroclone), 25 mmol/L HEPES, 100 U/mL penicillin and 100 U/mL streptomycin (Gibco BRL, Thermo Fisher Scientific) in a humidified 5% CO_2_ incubator at 37 °C. 

### 2.7. Pharmacological Treatments with EphA Inhibitor ALW-II-41-27 and Cell Viability Assays

Cells in their exponential growing phase were plated in 96-well plates (2000 cells/well). After 24 h, the cells were treated with 1:2 scalar dilutions (2000 - 31.25 nM) of EphA2 inhibitor ALW-II-41-27 (MedChemTronica, Sollentuna, Sweden) for 72 h or left untreated. The cells’ viability was analyzed using a Cell Titer-Glo^®^ luminescent cell viability kit (Promega Italia, Milano, Italy). The luminescence signal was detected using a Synergy HT luminometer (Biotek Instruments, Winooski, VT, USA) and analyzed using Gen5 v1.09 software (Biotek Instruments). The post-treatment viable cell proportions were obtained after normalization with the untreated controls and the concentrations inhibiting 50% of the cell population (IC50); their 95% confidence intervals were calculated using CalcuSyn Software (Biosoft, Cambridge, UK). The cell growth assays were performed by plating cells in their exponential growing phase into 12-well plates (10,000/well). After 24 h, the cells were treated with 1:5 scalar dilutions (2000 - 80 nM) for 72 h or left untreated. The cells were stained with 0.1% crystal violet (Sigma-Aldrich). The image acquisition was performed by using a Bio-Rad Chemidoc™ Touch Imaging System (Bio-Rad Laboratories) and the area occupied by the cells was quantified using Quantity One software version 4.6.5 (Bio-Rad Laboratories). The experiments were performed in triplicate and means; the SEM and *p* values (two-way ANOVA with Tukey ‘s multiple comparisons) were calculated using GraphPad Prism software version 8.0 (GraphPad Software, San Diego, CA, USA).

## 3. Results

### 3.1. EphA2 Expression in Bone Sarcoma Cell Lines from the Cancer Cell lines Encyclopedia 

**Figure 1:**
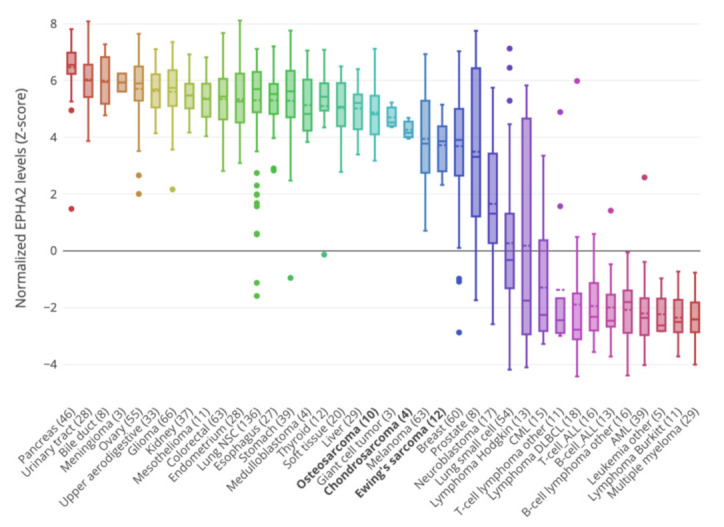
Distribution of EphA2 gene expression levels in the Cancer Cell Line Encyclopedia (CCLE). The box plot shows the Z-score-normalized EPHA2 levels in different cell lines divided by cancer type.

Recently, we showed that EphA2 down-regulation is implicated in the synergistic antitumor activity of pazopanib and trametinib in preclinical models of osteosarcoma. Moreover, the silencing of EphA2 expression led to impaired cell viability and cell migration in our models [19]. To further investigate the role of EphA2 as an effective molecular target in bone sarcomas, we took advantage of the public gene database on osteosarcoma cell lines and patient cohorts to validate our data in silico. As a first step, we explored EPHA2 gene expression levels in the whole Cancer Cell Line Encyclopedia (CCLE), which included 10 osteosarcoma, 12 Ewing’s sarcoma and 4 chondrosarcoma cell lines. As demonstrated in Figure 1, EPHA2 was highly expressed in the osteosarcoma (mean Z-score = 4.8), in Ewing’s sarcoma (mean Z-score = 3.9), and in chondrosarcoma (mean Z-score = 4.1) cell lines. The highest expression level was found in osteosarcoma Saos-2 (Z-score = 7.12), U2OS (Z-score = 5.50), and MG63 (z-score = 5.45) cells, followed by Ewing’s sarcoma SK-NEP-1 (Z-score= 5.28) RD-ES (Z-score = 5.15) and CADO-ES1 (Z-score= 4.71) cells. Finally, EPHA2 was remarkably expressed in three out of four chondrosarcoma cell lines: CAL-78 (Z-score= 4.68), Hs 819.T (Z-score= 4.16) and SW 1353 (Z-score = 3.95) (Figure 1). 

### 3.2. Correlation of EPHA2 Expression with Patients’ Characteristics and Clinical Outcome in Osteosarcoma

**Figure 2:**
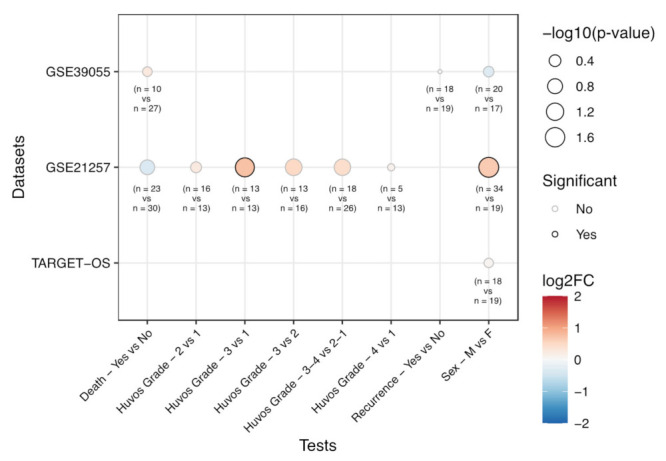
Bioinformatic analysis of EphA2 expression levels from public gene expression datasets on osteosarcoma samples, in relation to patient death status (sample sizes: Yes = 10, 23; No = 27, 30, for GSE39055, GSE21257 datasets, respectively), Huvos grade (sample sizes: G1 = 13, G2 = 16, G3 = 13, G4 = 5; GSE21257 dataset), disease recurrence (sample sizes: Yes = 18, No = 19; GSE39055 dataset), and sex (sample sizes: Male = 20, 34, 23; Female = 17, 19, 30, for GSE39055, GSE21257 and TARGET-OS dataset, respectively). The size of the dots is proportional to the significance of the statistical tests, while the color scale represents the log_2_ Fold Change (FC) of expression between the compared groups. Dots with a black border represent tests associated with a *p*-value lower than 0.05.

Next, we moved to the analysis of EPHA2 expression levels in relation to patients’ characteristics and clinical outcomes. This was performed using the 88 osteosarcoma samples included in the TARGET-OS project, 37 unique diagnostic biopsy specimens (GSE39055) and 34 pre-chemotherapy biopsies of osteosarcoma patients who developed metastases within 5 years, compared with 19 pre-chemotherapy biopsies of osteosarcoma patients who did not develop metastases in the same time interval (GSE21257), deposited in Gene Expression Omnibus (GEO). In the GSE21257 dataset, we found a significant (*p* = 0.035) EPHA2 higher expression rate in tumors with a higher Huvos grade compared to tumors with a lower Huvos grade (Figure 2 and Table 2). Furthermore, in the same dataset, EPHA2 was significantly upregulated (*p* = 0.019) in male compared to female subjects. With the limitation of the sample size, we did not find any significant association with survival outcomes.

### 3.3. Correlation of EPHA2 Expression and Clinical Features in Ewing’s Sarcoma

**Figure 3:**
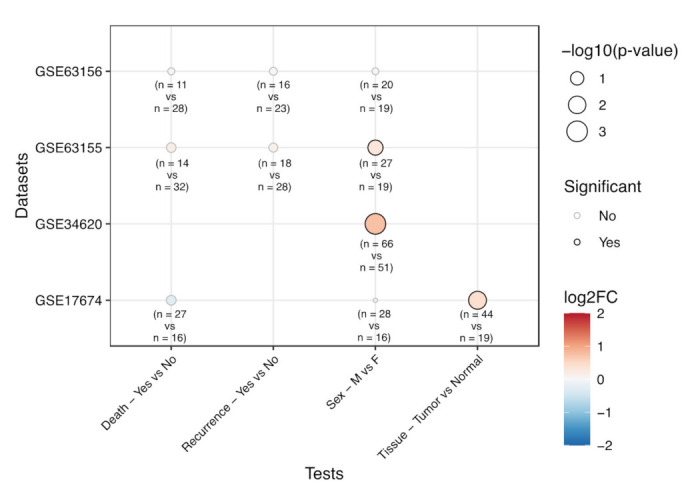
Bioinformatic analysis of EphA2 expression levels from public gene expression datasets on Ewing’s sarcoma samples, in relation to patient death status (sample sizes: Yes = 11, 14, 27; No = 28, 32 16, for GSE63156, GSE63155 and GSE17674 datasets, respectively), disease recurrence (sample sizes: Yes = 16, 18, No = 23, 28, for GSE63156 and GSE63155 datasets), sex (sample sizes: Male = 20, 27, 66, 28; Female = 19, 19, 51, 16, for GSE63156, GSE63155, GSE34620 and GSE17674 datasets, respectively) and tissue (sample sizes: Tumor= 44; Normal= 19, for GSE17674 dataset). The size of the dots is proportional to the significance of the statistical tests, while the color scale represents the log2 Fold Change (FC) of expression between the compared groups. Dots with a black border represent tests associated with a *p*-value lower than 0.05.

We took advantage of four microarray experiments deposited in GEO (GSE34620, GSE63155, GSE17674, GSE63156) and, using shinyGEO, we analyzed EPHA2 gene expression levels in relation to tissue type (tumor vs. normal), patients’ characteristics (age, gender) and clinical outcome (overall survival and recurrence data) in a total of 246 Ewing’s sarcoma patients. The analyses revealed a significantly (*p* < 0.01) higher EPHA2 expression in tumor samples compared to normal tissue and, in two datasets (GSE34620, GSE63155), a significant (*p* = 0.001 and *p* = 0.042, respectively) gene upregulation in tumors from male compared to female subjects (Figure 3). 

### 3.4. EPHA2 Expression in Chondrosarcoma and Correlation with Clinical and Molecular Features

**Figure 4:**
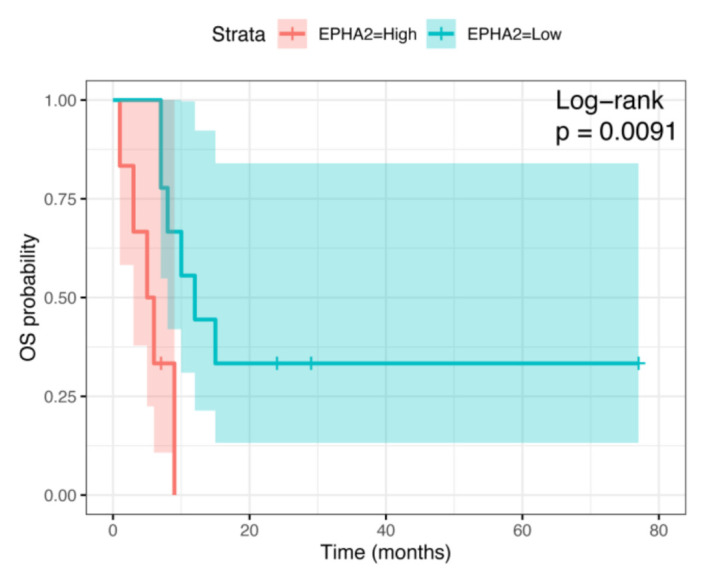
EPHA2 expression levels and overall survival (OS) probability in dedifferentiated chondrosarcoma group (*n* = 16).

**Figure 5:**
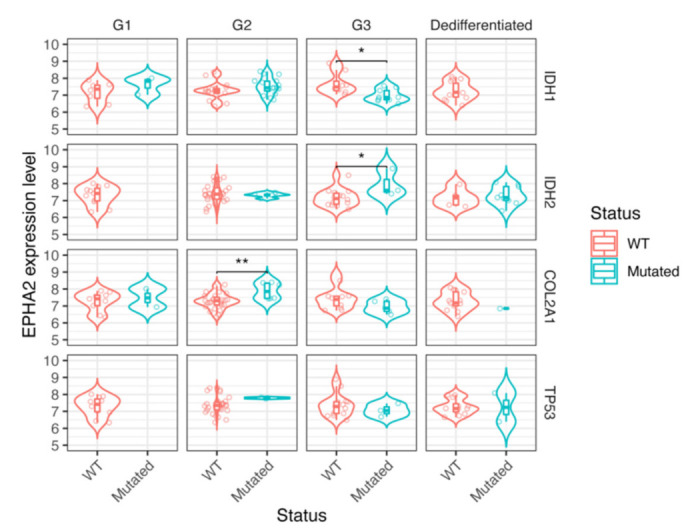
Boxplot showing association between chondrosarcoma mutational status and EPHA2 expression across different chondrosarcoma subtypes. The selected subtypes were dedifferentiated chondrosarcomas (*n* = 16), G1 chondrosarcomas (*n* = 17), G2 chondrosarcomas (*n* = 38) and G3 chondrosarcomas (*n* = 17) * *p*< 0.05; ** *p* < 0.01.

As mentioned above, we found EPHA2 gene expression in all but one of the four chondrosarcoma cell lines tested (Figure 1). As a further step, we took advantage of the mRNA expression dataset obtained by Nicolle R. et al., including samples from 102 chondrosarcoma patients [26]. Benign chondroid lesions (*n* = 8), included in their database, were excluded from our analyses, as well as those lesions with missing tumor grade data (*n* = 6). Patients affected by dedifferentiated chondrosarcomas (*n* = 16) with worse prognosis had significantly higher EPHA2 expression levels (*p* = 0.0091) compared to those with dedifferentiated chondrosarcomas, who experienced longer overall survival (Figure 4). Conventional chondrosarcomas had no significant association between prognosis and EPHA2 expression levels; however, there was a significant association between chondrosarcoma mutational status and EPHA2 expression. Indeed, the G2 chondrosarcomas with mutated COL2A1 demonstrated a higher EPHA2 expression with respect to their wild-type counterpart (Figure 5; ** *p* < 0.01). Considering G3 conventional chondrosarcomas, EPHA2 expression was lower in the IDH1-mutated samples when compared to the IDH1 wild-type samples (* *p* < 0.05), while the opposite was observed with respect to the IDH2 mutations (Figure 5; higher EPHA2 expression in IDH2-mutated samples; * *p* < 0.05).

### 3.5. EPHA2 Expression and Activation in Bone Sarcoma Patient-Derived Xenografts

**Figure 6:**
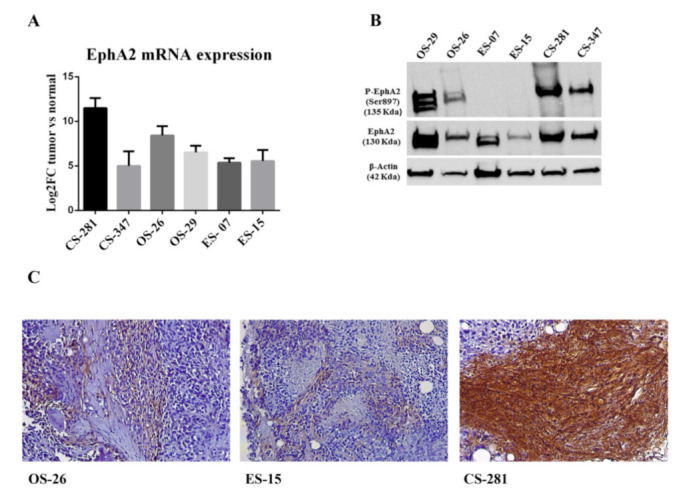
EphA2 expression in human bone sarcomas and patient-derived xenografts. (**A**) EphA2 mRNA expression in primary human tumors compared to normal counterparts, as obtained by quantitative real time PCR. (**B**) Western blot analysis of phosphorylated-EphA2 (Ser897), EphA2 and β-actin (housekeeping protein as loading control) as obtained from PDX protein extracts. Osteosarcoma (OS-26, OS-29); Ewing Sarcoma (ES-07,ES-15); Chondrosarcoma (CS-281, CS-347). (**C**) Immunohistochemistry showing EphA2 staining in PDXs.

We established six bone sarcoma PDXs in three generations of mice. According to the molecular and histopathological diagnosis, we selected two osteoblastic osteosarcomas, two Ewing’s sarcoma and two conventional chondrosarcoma PDXs. The quantitative real–time PCR analysis showed once again that EPHA2 mRNA levels were higher in primary tumor tissues than in surrounding normal tissues (Figure 6A). The western blot and immunohistochemistry analyses showed that EphA2 was also highly expressed in PDX tumor tissues at the protein level (Figure 6B,C). The activated phosphorylated form of EphA2 (p-EphA2^S897^) was displayed in osteosarcoma and chondrosarcoma but not in Ewing’s Sarcoma PDXs from our cohort (Figure 6B).

### 3.6. EphA2 Inhibitor ALW-II-41-27 Induced Dose-Dependent Anti-Tumor Effects in In Vitro Models

**Figure 7:**
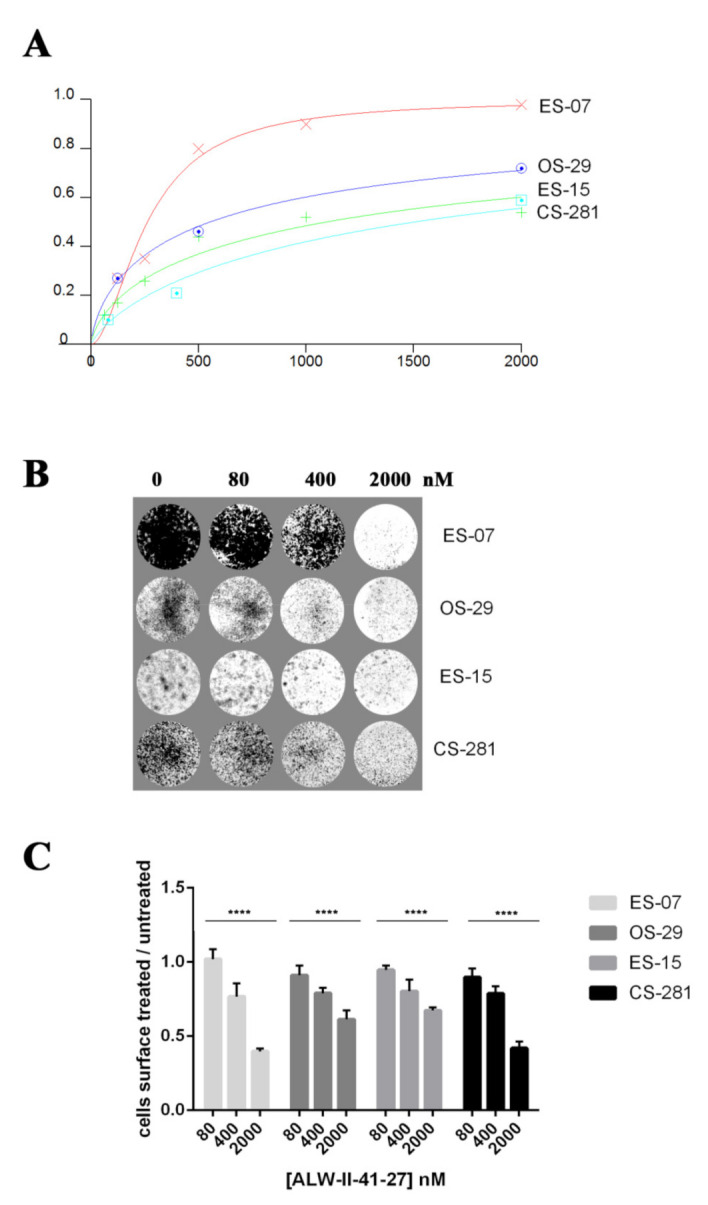
Dose-dependent antitumoral effects of the EphA2 inhibitor ALW II-41-27. (**A**) Cell viability assays and (**B**) cell growth assays after 72 h of treatment with scalar concentration of ALW II-41-27. (**C**) Quantification of the plate surface occupied by viable adherent cells, **** *p* < 0.0001.

In order to test the antitumoral effect of the EphA2 inhibitor ALW-II-41-27, we generated patient-derived in vitro models, starting with six primary tumor tissues (OS-29, OS-26, ES-7, ES-15, CS-281, and CS-347). Four out of six primary cell lines (OS-29, ES-07, ES-15, CS-281) were successfully established in adherent monolayer conditions. Treatment with scalar concentrations of ALW-II-41-27 induced a statistically significant dose-dependent inhibition of cell viability and cell growth of OS-29, ES-07, ES-15 and CS-281 cells, as demonstrated by the Cell Titer Glo Assays (Figure 7A) and crystal violet staining, respectively (Figure 7B,C). ES-07 were the most sensitive cells, followed by OS-29, ES-15 and CS-281 (*p* = 0.0167). The IC50s at 72 h were: 262 (95% confidence interval: 204–335), 552 (461–661), 1093 (773–1545), 1501 (766–2943), respectively.

## 4. Discussion

Our investigation of EPHA2 expression with bioinformatic analyses and PDXs highlights the involvement of EPHA2 in the tumor biology of the three main bone sarcoma histotypes: osteosarcoma, Ewing’s sarcoma and chondrosarcoma. In this specific setting, we still lack any substantial benefit from the innovation brought about by both target therapy and immunotherapy. In this context, EPHA2 may represent a step forward in both therapeutic settings. Indeed, innovative drugs have been developed targeting EphA2 both in the field of small inhibitors [27] and as drug conjugates [28] or EphA2-specific chimeric antigen receptor T cells (CAR-T; [20,21,22,23]). 

The search for tumor-specific targets has been a common feature through the whole field of oncology. Initially, research focused on proteins involved in key processes driving the proliferative advantage of each tumor histotype, achieving remarkable success in selected cancers, such as breast cancer (HER2) [29] or melanoma (b-RAF) [30]. Unfortunately, and despite international efforts, in bone sarcomas we could not identify any target such as KIT [31,32] or EGFR [33] through which to affect tumor biology in a significant way. So far, multi-kinase receptor inhibitors have demonstrated some improvements in osteosarcomas [3,4,5] and IDH-1/2 inhibitors in a minority of chondrosarcomas [8]. Recently, TK216, a specific inhibitor of the deranged transcriptional machinery of Ewing’s sarcoma, has shown preliminary hints of activity, but again in a minority of progressive patients [6]. Moreover, bone sarcomas have not benefitted from checkpoint blockade immunotherapy [34]. This scenario is the bottom line of the continuous efforts to identify new targets to selectively affect bone sarcoma tumor cells, with a “precision medicine” driven approach, trying to tailor each molecule to a specific histotype and disease setting.

In this context, we focused on EphA2, a well-known membrane-bound tyrosine kinase receptor that has already been shown to be involved in the proliferative advantage of several solid tumors [18]. In bone sarcomas, EphA2 has come to the fore for its relevance to Ewing’s sarcoma tumor angiogenesis [35] and metastasis [13]. Concerning osteosarcoma, EphA2 mitogenic-, migratory- and metastasis-promoting effects have been demonstrated by our group [19] and by Fritsche-Guenther et al. [36].

In this study, we performed bioinformatic analyses in several databases (fully listed in the Methods section) to look for EphA2 expression and correlation with patients’ characteristics and clinical outcomes in osteosarcoma, Ewing’s sarcoma and chondrosarcoma. 

The bioinformatic analysis of bone sarcoma cell line expression data (derived from CCLE) revealed high EPHA2 expression in osteosarcoma, Ewing’s sarcoma and chondrosarcoma; high expression levels were also found in other solid tumors, while low-to-null EPHA2 expression characterized hematological neoplasias. Hence, it seems that EPHA2 expression is not merely related to the cell of origin of the different tumor types (i.e., mesenchymal vs. epithelial). Indeed, one common feature of solid tumors is the need to hijack capable mechanisms through which to evade the primary tumor site and migrate to metastatic niches. With respect to blood cancers, solid tumors, these need to activate specific programs to orchestrate cell movement and migration, which often implies (for epithelial neoplasias) an epithelial-to-mesenchymal transition (EMT) [37,38]. Indeed, we could speculate that the high expression levels of EPHA2 observed in our analysis in aggressive epithelial cancers, such as pancreatic and gastric cancer, could reflect EMT activation in these histotypes. In fact, EphA2 is a key player in EMT and its involvement in this process has been specifically described in gastric cancer [39,40]. What is more, in our bioinformatic analysis, melanoma cells expressed EPHA2 at levels comparable to Ewing’s sarcoma and chondrosarcoma; in melanoma cells, EPHA2 expression has been linked to the shift from mesenchymal movement to amoeboid-like movement in migration and metastasis [41]. The ability of cancer cells to shuffle their movement type is an important mechanism of plasticity in cancer cell invasiveness [41,42,43,44]. Taken together, these data derived from CCLE bioinformatic analyses demonstrate high EPHA2 expression levels in solid tumors, including in our histotypes of interest (osteosarcoma, Ewing’s sarcoma and chondrosarcoma). 

Through the analysis of public gene expression databases including bone sarcoma patients, we confirmed in silico that EPHA2 is expressed at higher levels compared to normal tissues in Ewing’s sarcoma; that it is associated with Huvos grade in osteosarcoma; and that higher EPHA2 expression correlates with a worse prognosis in dedifferentiated chondrosarcoma. In both the osteosarcoma and Ewing’s sarcoma data sets, a significant difference in EPHA2 expression levels was found in male (higher expression) vs. female (lower expression) subjects. We could hypothesize that this might be related to the worse prognostic outcome of male patients with respect to female patients, reflecting the higher aggressiveness of tumors expressing EPHA2 at higher levels. However, for now, this could only be described as an association deserving attention, as we lack a causal explanation for this survival difference and its possible relationship with EPHA2 expression. Indeed, the physiological and biological reasons behind the different survival for male vs. female subjects in osteosarcoma and Ewing’s sarcoma are still largely unknown [45,46]. 

Moving from in silico analyses to our experimental in vitro and in vivo data, we provided evidence that EphA2 is highly expressed in selected primary tumor samples of bone sarcomas if compared to their normal counterparts; this was demonstrated in their respective PDX models generated in our laboratory in NSG mice. 

In particular, in both osteosarcoma PDX models, we showed EphA2 expression and phosphorylation at critical serine 897 (Ser897) residue, as a sign of oncogenic, non-canonical EphA2 pathway activation [14,15,16,47]. 

We also confirmed EphA2 expression in our Ewing’s sarcoma primary tumor samples and PDXs. However, in these two PDXs, we did not observe EphA2 phosphorylation at Ser897 (p-EphA2^S897^). In fact, previous work by Garcia-Monclús S. et al. [13] demonstrated heterogeneity among different Ewing’s sarcoma cell lines in terms of Ser897 phosphorylation. They demonstrated the role of EphA2 non-canonical pathway activation in the metastatic progression in Ewing’s sarcoma, displaying the more aggressive behavior (in terms of cell proliferation and migration) of cells bearing higher expression levels of p-EphA2^Ser897^ when compared to those with lower/no p-EphA2^Ser897^ [13]. Hence, considering the variability of p-EphA2^Ser897^ expression levels in Ewing’s sarcoma, it might be that with our limited sample size we could not capture this heterogeneity. Several other reasons might be hypothesized, but we speculate that this could also be due to the fact that our Ewing’s sarcoma models were derived from primary, localized tumor samples, and not from metastatic/relapsed lesions. 

Finally, for chondrosarcoma, we demonstrated abundant EphA2 expression at the tissue/protein level in our primary tumor samples and PDX models. This is a novel observation, which deserves particular attention due to the lack of both chemotherapeutic and innovative efficacious treatment strategies in chondrosarcoma. In the chondrosarcoma PDXs, the activated, phosphorylated form of EphA2, p-EphA2^S897^, was highly expressed, displaying functional non-canonical EphA2 pathway activation. 

Another attractive feature of EphA2 is its rather low physiological expression in healthy adult tissues [18], making EphA2 an interesting target with predictably few on-target, off-tumor side effects. Indeed, both in our bioinformatic investigation and in our qRT-PCR analysis of primary tumor and surrounding normal tissues, we demonstrated a significant differential expression of EPHA2 (higher expression in pathological tumor tissue vs. healthy tissues). Furthermore, we tested the sensitivity of EphA2 higher vs. lower expressing primary bone sarcoma cell lines, against the EphA2 inhibitor ALW-II-41-27. ALW-II-41-27 is a potent EphA2 inhibitor, which has displayed antitumoral activity in different tumor types and preclinical models. In particular, ALW-II-41-27 has been studied in non-small cell lung cancer [48,49], breast cancer [50], gastric cancer [51], colorectal cancer [52] and hepatocellular carcinoma [53]. However, this is the first time in which ALW-II-41-27 has been studied in bone sarcomas. We managed to establish six primary cell lines from our primary tumor samples, but only four out of these six were able to grow in monolayer-culture conditions in vitro. We observed a more profound effect on cell growth and viability in one Ewing’s sarcoma primary cell line, followed by osteosarcoma (intermediate sensitivity), another Ewing’s sarcoma (lower sensitivity) and chondrosarcoma primary cell lines. This represents an encouraging result for the chondrosarcoma histotype, in which EphA2’s role is a novel finding, as mentioned above, and which is particularly important given the current lack innovative therapeutic strategies. 

However, two caveats should be mentioned in this context: first, EphA2 is an important pattern recognition receptor for fungal beta-glucan [54] and its targeting might lead to impaired anti-fungal response. This could be a relevant issue for oncological patients, who are at relevant risk of developing opportunistic infections. Secondly, in the first three patients treated with EphA2-directed CAR-T cells, G2 pulmonary edema was reported, which could be an on-target, off-tumor effect considering that EphA2 is expressed in the adult lung epithelium [23]. 

Of course, our work features limitations, mainly due to the fact that, even among bone sarcoma subtypes, heterogeneity is a major issue. For instance, we provided data based on osteoblastic osteosarcoma models, but we cannot assume that our results could apply to other histotypes as well. Furthermore, pediatric osteosarcomas are somewhat different with respect to adult osteosarcomas [46]. In Ewing’s sarcoma – driven by several different specific balanced translocations – we might presume different levels of expression and involvement of this receptor pathway, based on Ewing’s sarcoma’s intrinsic heterogeneity [55,56]. Finally, chondrosarcomas display different grades of aggressiveness, which affect, for example, the IDH1/2 dependency of these tumors [8]. However, notwithstanding all these caveats, our findings provide a strong rationale upon which to develop future research targeting EphA2 in osteosarcoma, Ewing’s sarcoma and chondrosarcoma. Our PDXs could constitute a reliable platform to test different EphA2-targeting agents to strengthen preclinical data, in order to construct evidence for EphA2 targeting in clinical trials in different bone sarcoma histotypes. 

## 5. Conclusions

In conclusion, EphA2 is overexpressed in the three main bone sarcoma histotypes and EphA2 targeting with a small-molecule inhibitor demonstrated significant antitumoral effects in all the tested patient-derived bone sarcoma cell lines. In this specific setting, the array of pharmacological tools is rapidly enlarging and offers innovative types of drugs, both in the field of target therapies as well as in that of immunotherapies other than immune-checkpoint inhibitors. We believe that the data presented in this article warrant further studies in the field of osteosarcoma, Ewing’s sarcoma and, especially, chondrosarcoma, in which EphA2 pathway activation has not been previously reported and might represent a therapeutic opportunity for this disease.

## Figures and Tables

**Table 1:** Patients’ characteristics, tumor anatomical site, histotype and tumor grade and disease stage of the primary tumors that were analyzed and from which PDXs were generated.

PATIENT ID	Sex	Age	Anatomical Site	Histotype	Grade	Disease Stage
OS-026	M	67	Upper limb	Osteoblastic osteosarcoma	G4	Metastatic
OS-029	M	18	Lower limb	Osteoblastic osteosarcoma	G4	Metastatic
ES-07	F	19	Lower limb	Ewing’s sarcoma	High grade	Localized
ES-15	F	65	Lumbar spine	Ewing’s sarcoma	High grade	Localized
CS-281	F	58	Hip	Chondrosarcoma, conventional	G3	Locally advanced
CS-347	M	56	Upper limb	Chondrosarcoma, conventional	G2	Locally advanced

**Table 2:** Correlation of EphA2 expression level (Log2FC) with patient gender, Huvos grade, outcome and recurrence in osteosarcoma.

Dataset	Category	Test	EphA2 log _2_ FC	*p*-Value
GSE39055	Death	Yes vs. No	0.24	0.596
	Sex	Male vs. Female	-0.32	0.515
	Recurrence	Yes vs. No	-0.01	0.979
GSE21257	Sex	Male vs. Female	0.64	0.019
	Huvos Grade	2 vs. 1	0.24	0.474
	Huvos Grade	3 vs. 1	0.73	0.030
	Huvos Grade	3 vs. 2	0.50	0.081
	Huvos Grade	4 vs. 1	0.11	0.835
	Huvos Grade	3-4 vs. 2-1	0.43	0.088
TARGET-OS	Sex	Male vs. Female	0.06	0.628

## Data Availability

Data are available upon reasonable request.
